# The Role of Substance P in Secondary Pathophysiology after Traumatic Brain Injury

**DOI:** 10.3389/fneur.2017.00304

**Published:** 2017-06-28

**Authors:** Robert Vink, Levon Gabrielian, Emma Thornton

**Affiliations:** ^1^Sansom Institute for Health Research, University of South Australia, Adelaide, SA, Australia; ^2^Discipline of Anatomy and Pathology, Adelaide Medical School, University of Adelaide, Adelaide, SA, Australia

**Keywords:** substance P, traumatic brain injury, edema, intracranial pressure, brain oxygenation, sheep model

## Abstract

It has recently been shown that substance P (SP) plays a major role in the secondary injury process following traumatic brain injury (TBI), particularly with respect to neuroinflammation, increased blood–brain barrier (BBB) permeability, and edema formation. Edema formation is associated with the development of increased intracranial pressure (ICP) that has been widely associated with increased mortality and morbidity after neurotrauma. However, a pharmacological intervention to specifically reduce ICP is yet to be developed, with current interventions limited to osmotic therapy rather than addressing the cause of increased ICP. Given that previous publications have shown that SP, NK1 receptor antagonists reduce edema after TBI, more recent studies have examined whether these compounds might also reduce ICP and improve brain oxygenation after TBI. We discuss the results of these studies, which demonstrate that NK1 antagonists reduce posttraumatic ICP to near normal levels within 4 h of drug administration, as well as restoring brain oxygenation to near normal levels in the same time frame. The improvements in these parameters occurred in association with an improvement in BBB integrity to serum proteins, suggesting that SP-mediated increases in vascular permeability significantly contribute to the development of increased ICP after acute brain injury. NK1 antagonists may therefore provide a novel, mechanistically targeted approach to the management of increased ICP.

Traumatic brain injury (TBI) has been identified as one of the leading causes of death and disability in individuals less than 40 years of age in developed countries (1, 2). Despite the significance of this public health issue, there is currently no accepted therapy that can improve outcome (3), largely because the pathophysiological factors and their mechanistic interaction in the injury process have not been well characterized. In addition to the primary (mechanical) injury caused at the time of the traumatic event, secondary injury factors play a major role in resultant neuronal cell death that results in lifelong disability experienced by many survivors. While a number of secondary injury factors have been identified (4, 5), water accumulation, or brain edema, has been recognized as being closely associated with patient outcome (6). Indeed, up to half of all deaths following TBI having been attributed to brain edema (7, 8). A number of treatment strategies have been introduced to relieve edema-associated brain swelling (9); however, these target the end result of the edematous process [increased intracranial pressure (ICP)] rather than the cause. These strategies include administration of hyperosmotic agents and barbiturates, hypothermia, hyperventilation, cerebrospinal fluid (CSF) drainage, and decompressive craniotomy (10). However, significant improvements in patient mortality and morbidity have not been observed with these interventions, largely because they do not attenuate the specific mechanisms associated with edema formation after TBI.

Increased ICP can locally compress tissue, reduce cerebral perfusion, reduce brain oxygenation with resultant hypoxia and ischemia, result in brain herniation and, in severe cases, cause death. For ICP to increase after TBI, the volume within the confines of the skull must increase either through vasodilation, hemorrhage, increased CSF production, reduced CSF reabsorption, or edema. If ICP is increased because of edema, it follows that the total fluid volume of the cranial vault must have increased, and the only source of this water can be the vasculature (11, 12). Water movement from the cerebral vasculature to the brain parenchyma is known as vasogenic edema and has been well described as a significant component of early edema formation after TBI (6). Moreover, vasogenic edema is known to be permissive for subsequent cytotoxic edema (13), which then initiates a feedback loop that further drives increases in vasogenic edema and consequently ICP (12). Vasogenic edema requires that there is increased blood–brain barrier (BBB) permeability to serum proteins. A net movement of water from the vascular compartment then follows extravasation of blood plasma proteins into the brain parenchyma, leading to a disruption of fluid homeostasis. Given the increase in the volume of the brain tissue under these circumstances, ICP rises and negatively influences patient outcomes (14). In addition, the loss of barrier integrity following acute injury to the brain allows peripheral immune cells to cross the barrier and further contribute to and exacerbate the inflammatory processes within the brain (15).

Increased BBB permeability after TBI with subsequent edema formation has been recently linked to substance P (SP) release (16, 17). Moreover, the increased BBB permeability and edema formation after acute brain injury, together with the associated increased ICP, was reduced by administration of a SP, NK1 receptor antagonist (17–19). Accordingly, the current review focuses on the role of SP in TBI, particularly in relation to edema formation and increased ICP.

## Substance P

Substance P is peptide of 11 amino acids and belongs to the large tachykinin peptide family containing over 40 tachykinins, including neurokinin A, neurokinin B, neuropeptide-γ (NPγ), and the recently identified hemokinin 1. Originally identified in the 1930s by von Euler and Gaddum for its potent smooth muscle and hypotensive properties (20), SP is now known as a neurotransmitter that is released from primary afferent neurons in both the peripheral and central nervous system, as well as from non-neuronal cells such as inflammatory and endothelial cells (21, 22). In the nervous system, SP is localized to nuclei such as the substantia nigra and the medial amygdaloid nucleus, as well as to capsaicin-sensitive sensory neurons (23) where it is released in response to stimulation of the transient receptor potential channels by mechanical stimulation, temperature, pH changes, and ligand binding (24). Following release, SP can bind to tachykinin NK receptors to exert direct postsynaptic actions as a neurotransmitter or modulate other non-neuronal targets (22). The NK receptors are 7-transmembrane domain, G-protein-coupled receptors, with three, known as the NK1, NK2, and NK3 receptor, having been identified to date (21). Each of the tachykinin neuropeptides is able to bind all three receptor types depending on receptor availability and concentration of the neuropeptide, indicating that there is a degree of cross-reactivity among the receptors (21). SP normally has the highest affinity for the NK1 receptor, which given the predominance of the NK1 receptor in the adult brain (25) makes SP the tachykinin of particular interest in the context of CNS injury.

### Synthesis

Two genes exist that are relevant to tachykinin synthesis, namely the preprotachykinin (PPT) A gene and the PPTB gene. Four different forms of mRNA are expressed through alternative splicing from the PPTA gene (26), the α and δ forms exclusively encoding for the synthesis of SP, while the β and γ forms encode synthesis of SP, as well as NKA, neuropeptide K (NPK), and NPγ; NPK and NPγ are elongated forms of NKA. In the brain, αPPTA expression is predominant, while in the peripheral tissues, βPPTA and γPPTA mRNAs are abundant (27). The PPTB gene encodes neurokinin B (22).

Similar to all neuropeptides, SP is synthesized on ribosomes that are exclusively present in the cell body. The mRNA encoding the tachykinin is initially translated into a larger protein precursor from which SP is subsequently released by the actions of proteases called convertases. Cleavage points for the convertases are doublets of cationic residues (26). After release, the actions of tachykinins are terminated by diffusion away from the receptor site or degradation by extracellular peptidases, the slow nature of these processes accounting for their prolonged effects (28).

### Metabolism

Several enzymes are associated with SP metabolism including neutral endopeptidase (NEP) (29), angiotensin-converting enzyme (ACE) (30), SP-degrading enzyme (31), post-proline endopeptidase (32), dipeptidyl aminopeptide IV (33), cathepsin-D (34), and cathepsin-E (35). The individual cellular localization of NEP and/or ACE suggests that these enzymes are most likely responsible for the *in vivo* cleavage of SP (36), albeit that all of these enzymes have been shown to cleave the tachykinin *in vitro*. Hydrolysis of SP by both ACE and NEP removes the carboxyl terminal required for binding to the tachykinin receptors (30). NEP hydrolyzes SP in the peripheral tissues, brain, and spinal cord (37–39) with ACE-degrading SP in the plasma, CSF, and the substantia nigra (40), as well as contributing to the degradation of peptide fragments released by NEP.

### Localization

Immunohistochemistry has shown that SP is present in the diencephalon, telencephalon, rhinencephalon, hippocampus, basal ganglia, pons, amygdala, hypothalamus, septal areas, myelencephalon mesencephalon, metencephalon, and spinal cord (41). SP immunopositive nerve fibers are common in most autonomic ganglia (42–44) and are detected in trigeminal and dorsal root ganglia (45, 46) as well as in intrinsic neurons of the gut (47). It is thought to play a modulatory role in the autonomic ganglia, the best characterized response being observed in guinea pig inferior mesenteric ganglion where SP mimics a slow depolarization that can be evoked by repetitive afferent nerve stimulation (48). Peripheral inflammation has been shown to increase SP immunoreactivity in the superficial layers of the spinal cord (49) and increase release of SP (50). Damage to neurons or their intense activation also induces neuropeptide gene expression leading to alterations in neuropeptide biosynthesis (51). Specifically, during noxious stimulation or neurogenic inflammation in the periphery, there is an upregulation (28) of PPT mRNA expression (52) and NK1 receptor mRNA (53).

## Neurogenic Inflammation

Sensory nerve fibers positive for both SP and calcitonin gene-related peptide (CGRP) are found surrounding most blood vessels throughout the body. In particular, cerebral arteries have a rich supply of sensory neurons, suggesting that they have a role as mediators of the inflammatory process following injury. The release of these neuropeptides, including SP, is neurally elicited and results in a painful local inflammatory response known as neurogenic inflammation, characterized by increased vascular permeability, protein extravasation, mast cell degranulation, and vasodilation (54). These changes in vascular permeability and in blood vessel diameter result in localized swelling of the tissue (54). Also occurring are tissue-specific responses to neuropeptide stimulation including constriction of the bronchioles in the airways and contraction and/or relaxation of the smooth muscle in the bladder. SP is also widely considered to be the most active mediator of neurogenic inflammation, even though it is well known that other neuropeptides such as CGRP are involved. Nonetheless, CGRP potentiates the effects of SP by enhancing the bioavailability of SP through competition with SP for metabolism by endopeptidases and by increasing the expression of the NK1 tachykinin receptor (55). Neurogenic inflammation in itself also leads to an increase in the PPTA and NK1 receptor mRNA transcript (28), which encodes SP and its primary receptor, respectively.

A role for classical inflammation in the pathophysiology of secondary injury following TBI is well known (15); however, brain neurogenic inflammation has remained relatively unexplored until recently. First characterized in peripheral tissue, neurogenic inflammation has now been well described in a number of studies following acute brain injury (3, 16, 56). Its occurrence in the CNS was first demonstrated when electrical or chemical stimulation of the Dura mater, or acute capsaicin administration (a TRPV1 agonist), produced a local neurogenic inflammatory response in the form of increased protein extravasation that was not observed in the brain parenchyma itself or in the Pia mater (57). Subsequently in stroke, activation of endothelial NK1 receptors on blood vessels was shown to contribute to cerebral edema (58). Administration of SP was then shown in rats to produce a profound increase in plasma protein extravasation in the Dura mater, which was blocked when an NK1 receptor antagonist was administered and exacerbated by administration of either NEP or ACE inhibitors (59). In studies of TBI, inhibition of posttraumatic neurogenic inflammation by prior depletion of sensory neuropeptides using chronic capsaicin pretreatment attenuated increased BBB permeability, and the development of edema and functional deficits (60, 61), with subsequent studies demonstrating that ACE inhibitors exacerbated histological damage and functional deficits after TBI (62). Further studies in stroke established that reversible ischemic stroke resulted in increased brain perivascular immunoreactivity to SP with associated edema formation (63), while decreased SP immunoreactivity in association with increased NK1 immunoreactivity in both rat and human spinal cord injury suggested a role for neurogenic inflammation in this form of CNS injury (64, 65). Finally, activation of the multimodal TRPV1 receptor that is linked to SP release initiates neurogenic inflammation and is associated with increased BBB permeability, an effect abolished by the TRPV1 antagonist capsazepine and by an NK1 antagonist (66). Collectively, these data provide strong support that neurogenic inflammation involving the release of SP can occur within the setting of acute CNS injury.

## SP in TBI

Our own studies have shown that SP release is a ubiquitous feature of TBI and is associated with marked increases in BBB permeability, edema formation, and the development of functional deficits (56). Specifically, an increase in cerebral perivascular SP is observed following TBI as early as 5 h after TBI and persisting for at least 24 h following trauma (17). In human postmortem TBI tissue, the increased SP immunoreactivity colocalized with APP in perivascular nerve fibers suggesting that injury to these perivascular neurons was associated with SP release (67). The authors also reported that increased SP was apparent in cortical neurons and astrocytes, similar to observations made in the rodent models (17). SP mRNA levels as determined by PCR analysis remained elevated until at least 3 days posttrauma (68), suggesting persistent synthesis and release over this time frame. Moreover, serum levels of SP were elevated after TBI, with significant increases observed in both experimental (17) and human TBI, the latter having been associated with increased severity and mortality in patients (69). As discussed earlier, when SP catabolism is inhibited by the administration of an ACE inhibitor, further increases in SP immunoreactivity is observed together with an exacerbation of injury and neurological dysfunction (62).

Such increases in SP levels following trauma have been associated with increased permeability of the BBB and the formation of cerebral edema (12). Specifically, increased perivascular SP immunoreactivity after TBI colocalized with increased extravasation of Evan’s blue dye, a marker of increased BBB permeability (17). The authors proposed that where SP was bound to a vascular endothelial cell, BBB permeability to vascular protein was increased. This increased BBB permeability to proteins was associated with the development of cerebral vasogenic edema, together with the development of persistent motor and cognitive deficits (17).

Further evidence supporting a role for SP in neurogenic inflammation after TBI has been obtained using NK1 antagonists (3). For example, the NK1 tachykinin receptor antagonist *N*-acetyl-l-tryptophan (NAT) attenuated increased BBB permeability, cerebral edema, and functional deficits when administered at 30 min after TBI (17). Attenuation of BBB permeability toward Evan’s blue was dose dependent and used to determine the optimal dose. The therapeutic window of the antagonist was established as 12 h with rats administered with the compound at such delayed time points still demonstrating reductions in neuronal injury and an improvement in functional outcome (70). However, only a membrane permeable form of the drug was effective at these later time points, suggesting that the efficacy at delayed time points was dependent on central penetration of the compound. These studies also established that inactive enantiomers of the active ligands were ineffective irrespective of the time point, emphasizing that neuroprotective efficacy was dependent on actual binding to the NK1 receptor and the inhibition of its activity.

Most experimental studies are confined to male animals, largely to avoid the confounding effects of gender related hormones. However, the efficacy of the NK1 antagonists in TBI has also been demonstrated in female animals (71). Specifically, increased SP immunoreactivity was again apparent after diffuse TBI and the NK1 antagonist, NAT, reduced BBB permeability to albumin, reduced axonal injury, and significantly improved functional outcome. Moreover, it reduced edema formation at 24 h after TBI by more than 80%. In related studies involving reversible ischemic stroke, administration of the NK1 antagonist at 4 h after stroke onset resulted in reduced BBB permeability and edema formation at 24 h, plus improved functional outcome over 1 week (18). Indeed, treatment with the NK1 antagonist was more effective than neuropeptide depletion with capsaicin pretreatment (72) and importantly did not reduce the effectiveness of tissue plasminogen activator (tPA) treatment (73). When combined with tPA, the NK1 antagonist actually appeared to stabilize the BBB and extend the therapeutic window of the tPA treatment, which in clinical scenarios has been limited to 4 h.

## ICP and Brain Oxygenation after TBI

Various animal models have been developed to reproduce aspects of the pathophysiology observed clinically after TBI, with rat models being the most widely used in experimental neurotrauma because they are considered cost effective and have readily available outcome measures (74). Disappointingly, however, treatments that have proven to be neuroprotective in these rodent models have not successfully translated to the clinical environment, thus emphasizing the importance of validating promising therapeutic agents in arguably more clinically relevant large animal models before progressing to clinical trials. Accordingly, we have developed a large animal model of diffuse TBI using sheep that reproduces consistent changes in ICP and brain tissue oxygenation (P_bt_O_2_) that are more representative of the clinical situation (75, 76). The use of a large animal, ovine model of TBI delivers a number of advantages that are not present in the more commonly used rodent models of TBI. First, there is the presence of a gyrencephalic brain with large white matter domains as opposed to a lissencephalic brain. The presence of gyri alters the mechanical response of the brain to TBI, while the large white matter domains alter the edema response. Sheep also have a significant tentorium cerebelli thus separating the brain into supratentorial and infratentorial compartments, similar to that observed in humans (77). These differences in cerebral folding, gray matter/white matter distribution, and brain compartmentalization may therefore contribute to the inability of some groups to consistently produce posttraumatic ICP responses in rats in the absence of mass lesions or hypoxia (78). Moreover, unlike rodents, sheep have remarkably similar ICP and P_bt_O_2_ values to humans, with normal ICP in the sheep between 6 and 9 mm Hg and P_bt_O_2_ being above 40 mm Hg, as well as the similar responses in these important physiological variables after TBI (75). Finally, large animals are amenable to using the same neurosurgical techniques and surgical instrumentation as that used clinically, which is also an advantage.

**Figure 1:**
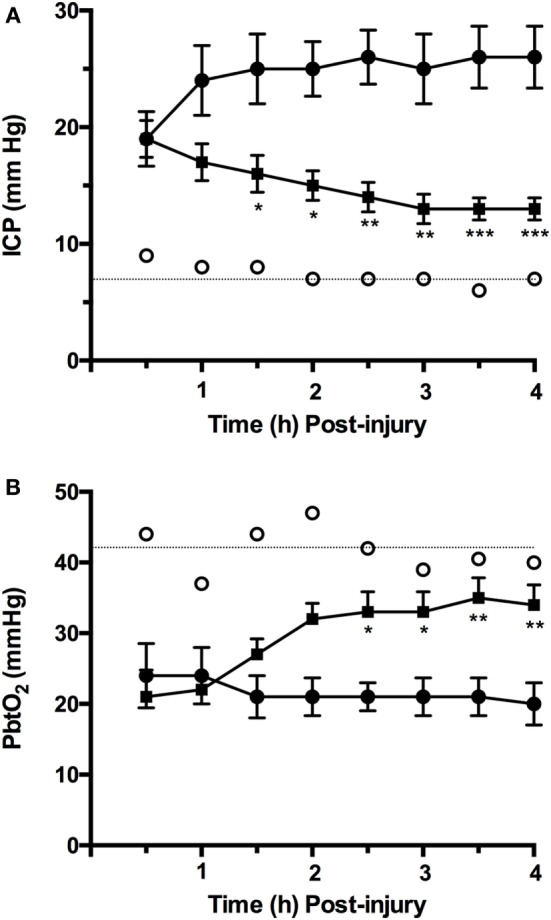
Time course of changes in **(A)** intracranial pressure (ICP) and **(B)** P_bt_O_2_ following moderate to severe diffuse traumatic brain injury in sheep and treatment with an NK1 antagonist [adapted from Ref. (19, 75)]. Briefly, 2-year-old isoflurane anesthetized merino sheep were injured using the humane stunner and monitored for ICP and P_bt_O_2_. *N*-acetyl-l-tryptophan (NAT: 2.5 mg/kg i.v.) was administered at 30 min after injury. ○ = sham (uninjured) animals (*n* = 9); • = vehicle (saline) treated animals (*n* = 9); ▪ = NAT-treated animals (*n* = 10). *p < 0.05; **p < 0.01; ***p < 0.001 versus vehicle-treated animals (two-way ANOVA followed by Bonferroni *post hoc* tests).

We have previously shown that ICP in moderate/severely injured sheep increases from control values of approximately 7 mm Hg to above 20 mm Hg within 1 h of the injury [(75); Figure 1A] and remains at those elevated levels in the hours that follow. This increase in ICP is consistent with the presence of vasogenic edema formation previously reported in diffuse TBI, and peaking between 4 and 6 h after injury (79, 80). Indeed, in the sheep brain, there was significant albumin extravasation at these early time points after TBI, confirming the presence of a more permeable BBB (80). When the NK1 antagonist, NAT, was administered at 30 min postinjury, there was a significant and sustained decline in ICP (19). Focusing on the time course of those previously reported changes [(19); Figure 1A], the NK1 receptor antagonist significantly reduced ICP by 32% within 3.5 h of administration, whereas ICP continued to increase by a further 36% in vehicle-treated animals. By 4 h after injury, ICP in the NK1-treated animals was half of vehicle-treated animals (*p* < 0.001) and approaching normal values (19).

The reoxygenation of brain tissue after TBI is an important part of effective therapeutic intervention, with restoration of aerobic energy metabolism essential to enable damaged tissue to recover. In the ovine model of diffuse TBI, injury typically results in a significant fall in P_bt_O_2_ to less than 50% of normal values, a reduction that persisted over the next few hours [(75); Figure 1B]. Administration of an NK1 receptor antagonist increased P_bt_O_2_ to more than 80% of control values by 4 h after TBI (81), consistent with the close relationship that has been previously described between ICP and P_bt_O_2_ after TBI (75). This improved oxygenation of the brain following administration of the NK1 antagonist may, in part, account for the improved neuronal cell survival and associated improvement on functional outcome previously reported (3).

A number of other experimental compounds have been successfully used to reduce edema formation in experimental TBI studies, including more recently progesterone and magnesium (4, 82), both of which have been unsuccessful in clinical trials to date (83, 84). Notably, when tested in our sheep model of TBI, our preliminary results also showed that both compounds had little effect on ICP or P_bt_O_2_ after TBI (unpublished results). The hyperosmotic compound mannitol is used clinically in the management of increased ICP following TBI, although considerable variability has been observed in both the ICP (9, 85) and P_bt_O_2_ response (86) in these clinical studies. The mechanism of action of mannitol is to draw water out of brain tissue into the vasculature where its presence has increased the osmotic pressure. This does not attenuate the potential mechanisms driving an increase in ICP, where the BBB after TBI is more permeable to proteins. There is also the potential that mannitol may actually cross into the brain parenchyma through the more permeable BBB, increasing the brain osmotic pressure and causing water influx with a rebound increase in ICP (87). By contrast, the mechanism of action of the NK1 receptor antagonist involves reducing BBB permeability and inhibiting the development of vasogenic edema (17), thus eliminating any possibility for a rebound increases in ICP.

## Mechanism of Action

**Figure 2:**
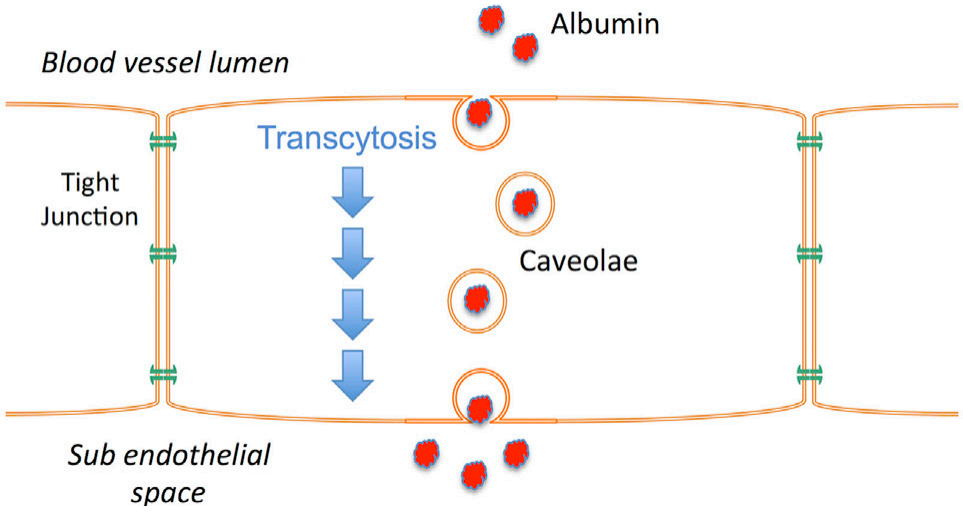
Schematic illustrating the process of transcytosis where vascular proteins such as albumin are transported across the endothelial cells of the blood–brain barrier *via* caveolae. The creation of a protein osmotic gradient then drives water influx from the vasculature to the brain via aquaporin channels. NK1 receptors are present in caveolae.

Vasogenic edema occurs when a BBB with increased permeability to vascular protein facilitates the influx of proteins such as albumin into the brain; vascular water subsequently follows down the osmotic gradient that has been created. A number of events have been associated with both the early and late BBB disruption described after TBI, including classical inflammation, activation of matrix metalloproteinases, metabolic imbalances, and breakdown of tight junction proteins (88–90). However, the increased movement of proteins across the BBB does not require the physical breakdown of the barrier or the tight junctions, but rather can occur *via* caveolae in a process known as transcytosis (91). Indeed, in the hours following TBI, the tight junctions of the BBB have been shown to be intact (92, 93) as opposed to the caveolae that are upregulated (93). Increased caveolin-1 expression, a major constituent of caveolae, is thus thought to reflect an increase in albumin transcytosis after TBI (93), and account for the vasogenic edema that ensues (Figure 2). NK1 receptors have been shown to be located in caveolae (94–96), suggesting that their activation may play a role in regulating transcytosis. The fact that NK1 antagonists reduce BBB permeability after TBI, at a time when the tight junctions are intact and the BBB is more permeable to albumin, supports this suggestion. Thus, the release of perivascular SP after TBI activates NK1 receptors, including those localized to caveolae. This activates transcytotic albumin transport from the vasculature to the brain parenchyma, creating a protein osmotic gradient that drives water entry through aquaporin channels with subsequent edema formation. By inhibiting transcytosis, the NK1 antagonists attenuate the development of an osmotic gradient and negate the requirement for water movement from the vasculature to the brain. Without increased volume from vascular-derived water, there will not be an edema-associated increase in ICP, and any water that has accumulated in the brain will now be able to efflux *via* aquaporin channels (97).

## Conclusion

NK1 antagonists reduce posttraumatic ICP to near normal levels within a few hours of drug administration. They also restore P_bt_O_2_ to normal levels in the same time frame, confirming an association between ICP and P_bt_O_2_ after TBI. The effects of NK1 antagonists on these parameters are more consistent and generally superior to mannitol, without the risk of rebound increases in ICP, and significantly better than the experimental treatment strategies progesterone and magnesium, which are ineffective. We posit that SP-mediated increases in protein transcytosis increases vascular permeability, significantly contributing to the development of increased ICP after acute brain injury. Administration of NK1 antagonists reduces this protein transcytosis, eliminating the driver for vasogenic edema and thus reducing increased ICP. Accordingly, the NK1 antagonists warrant further investigation as a novel therapeutic approach to the management of increased ICP.

## Author Contributions

RV drafted the manuscript and prepared all of the figures. LG and ET contributed to Figure 1 and drafted sections of the manuscript.

## Conflict of Interest Statement

The authors declare that the research was conducted in the absence of any commercial or financial relationships that could be construed as a potential conflict of interest.
